# Construction of Balanced *n*-ary Designs via Complete Multiset Spaces

**DOI:** 10.21203/rs.3.rs-8595082/v1

**Published:** 2026-04-21

**Authors:** Sudesh K. Srivastav, Apurv Srivastav

**Affiliations:** 1 Department of Biostatistics and Data Science, Tulane University, New Orleans, USA; 2 Department of Electrical and Computer Engineering, Center of Bioinformatics and Computational Biology, University of Delaware, Newark, USA

**Keywords:** Balanced *n*-ary design, Multiset design, Pairwise concurrence, Information matrix, Optimal design, 62K10, 05B05

## Abstract

We study balanced n-ary block designs that allow repeated treatments within blocks, motivated by applications in dose-response and repeated-measure experiments. We introduce a master (*k* + 1)-ary design with explicit replication and pairwise concurrence parameters. Systematic symmetry-preserving deletions generate infinite families of lower-arity balanced designs, for which explicit formulas for replication numbers, structured expressions for pairwise concurrences, and the resulting information matrix under the fixed-effects model are obtained.

These results establish a rigorous link between combinatorial *S_v_*-symmetry and statistical optimality. Simulation studies show that the proposed multiset-based designs, by maintaining equireplication, yield lower contrast variances than random multiset arrangements with unequal replication numbers. Potential applications include doseresponse studies, resampling-based simulations, and repeated-measures experiments.

## 1 Introduction

Block designs allowing repeated elements within blocks arise naturally in experimental settings involving multiple applications of the same treatment or dosage. Such designs were first considered by Tocher [1] and later formalized as balanced n-ary designs, in which each element may appear up to n−1 times within a block. Here, n denotes the number of possible values for treatment multiplicities (0, 1,…,n−1), not the number of treatments (v) or block size (k). Balanced incomplete block designs are recovered as the special case n=2. Throughout, we use the convention that an n-ary design permits multiplicities in 0, 1,…,n−1; thus a design allowing multiplicity up to k is referred to as (k+1)-ary.

A balanced n-ary design is a collection of b blocks, each a multiset of size k from a set of v elements, such that: (i) each element appears exactly r times across all blocks (replication number), (ii) no element appears more than n−1 times in any block, i.e., multiplicities lie in {0, 1,…,n−1}, and (iii) for any two distinct elements i and j, the pairwise index

$$\lambda (i,j)=\sum_{\text{blocks}}m_{i}m_{j}$$

is constant across all unordered pairs, and this common value is denoted by λ. The above definition is consistent with the multiset design framework of Shah and Dey 4 and subsequent generalizations by Billington [5; 6], in which balance is defined via constant weighted pairwise concurrences, not classical co-occurrence. Throughout, an “n-ary” design is understood to allow multiplicities in {0, 1,…,n−1}; in particular, the master multiset design D(v:k) introduced in Section 2 is (k+1)-ary. Derived subdesigns enforce smaller admissible multiplicity ranges as specified in Section 3. As in classical balanced incomplete block designs, the parameters satisfy bk=vr, and the constant pairwise index λ generalizes the usual concurrence parameter.

The systematic construction of balanced designs traces back to the Method of Difference introduced by Bose [2], which revolutionized the field by providing algebraic techniques to generate balanced incomplete block designs (BIBDs). While subsequent developments by Dey [3] and Shah and Dey [4] extended these concepts to n-ary structures, many traditional methods still necessitate the complex search for initial blocks or difference sets. In contrast, the master design framework proposed here exploits the inherent $S_{v}$-symmetry of the full multiset space D(v:k) to achieve balance without searching for initial blocks or difference sets. By shifting the constructive paradigm from algebraic seed blocks to symmetry-preserving reductions of a master design, the approach yields a unified framework that is computationally explicit and theoretically robust.

Balanced n-ary designs, including ternary and quaternary designs, have been studied extensively (Billington [5], [6]). Construction methods and existence results for specific block sizes and arities have been developed by Shah and Dey [4], and Donovan [7], among others. Recent developments include nested balanced n-ary designs (Dey and Midha [8]; Sharma and Fanta [9]) and recursive constructions for t-designs (Phad and Pawar [10]). Closely related are the multiset designs introduced by Assaf et al. [11], which allow repeated elements within blocks subject to prescribed balance conditions. While these frameworks provide considerable flexibility, many existing constructions focus on specific arities or parameter ranges and typically allow repeated blocks. Moreover, they often establish balance primarily through element-frequency conditions, without furnishing a single underlying structure from which whole families of balanced designs of varying arity can be generated in a uniform way.

In this paper, a unified multiset viewpoint is adopted to show that the collection of all *distinct* multisets of size k drawn from a v-set forms a balanced (k+1)-ary design with explicitly computable parameters. Unlike constructions based on difference sets or recursive embeddings, the present approach begins with the complete multiset space and applies symmetry-preserving deletions, eliminating initial-block searches and yielding closed-form expressions for replication and concurrence parameters. This master design then generates several infinite families of balanced n-ary designs of lower arity via systematic block deletion, while guaranteeing block distinctness and retaining closed-form parameter expressions. As astructural consequence of the underlying $S_{v}$-symmetry, the deletion schemes unify different arities, satisfy the generalized Fisher inequality b≥v (Preece [12]), and provide analytically tractable designs with explicit contrast variances under fixed-effects models. Unlike Assaf et al. [11], who allow repeated blocks and variable block sizes, our construction uses the complete space of distinct multisets as a master design, ensuring both block distinctness and explicit symmetry. This structural viewpoint appears to be absent in the existing multiset-design literature.

The remainder of the paper is organized as follows. In Section 2, we describe the multiset representation of blocks and derive the basic design parameters of the resulting balanced (k+1)-ary design. Section 3 develops systematic deletion schemes that generate balanced designs of lower arity and establishes their corresponding design parameters. Concluding remarks and directions for further research are given in Section 4.

## 2 Construction of Balanced (k+1)-ary Designs

### 2.1 Multiset representation

Let

E={1, 2,…,v}

be a set of v elements. A block is defined as a multiset of size k, represented by a vector

$$\left(x_{1},x_{2},\ldots ,x_{v}\right),$$

where $x_{i}\geq 0$ and

$$\sum_{i=1}^{v}x_{i}=k.$$

Here $x_{i}$ denotes the multiplicity of element i in the block. Since $x_{i}\in 0, 1,\ldots ,k$, any such design is (k+1)-ary. Each such vector encodes a block by its multiplicity pattern over the v treatments. Let D(v:k) denote the design consisting of all distinct multisets of size k drawn from E. When needed, we write D(v:k,b) to indicate explicitly that the design has b blocks.

### 2.2 Enumeration of blocks

The number of nonnegative integer solutions to

$$\sum_{i=1}^{v}x_{i}=k$$

is

b=v+k−1k.

Thus, D(v:k) contains exactly b distinct blocks. This enumeration follows from the classical stars-and-bars argument, which counts the number of non-negative integer solutions to

$$x_{1}+\cdots +x_{v}=k.$$

Each such solution corresponds uniquely to a multiset block of size k drawn from v treatments.

### 2.3 Structural design parameters

Beyond enumeration, the complete symmetry of D(v:k) under permutations of the element labels ensures uniform replication and pairwise concurrence. These properties are formalized in the following theorem.

**Theorem 2.1** (Existence and design parameters). *Let* v≥2 *and* k≥2. *The design* D(v:k) *consisting of all distinct multisets of size* k *drawn from a* v-*set exists and forms a balanced* (k+1)-*ary design with parameters* (v,b,r,k,λ) *given by*

b=v+k−1k,r=v+k−1k−1,λ=v+k−1k−2.

*Proof*. Each block of D(v:k) is a nonnegative integer vector $\left(x_{1},\ldots ,x_{v}\right)$ satisfying $\sum_{i=1}^{v}x_{i}=k$. The number of such vectors is b=v+k−1k, and all blocks are distinct by construction.

Fix an element i. The number of blocks in which i appears exactly s times equals v+k−s−2k−s. Hence,

r=∑s=1ksv+k−s−2k−s=v+k−1k−1,

using a standard binomial summation identity (see Appendix A and, for example, Gould [14]; Riordan [15]). This expression is consistent with the symmetry argument that, among all distinct multisets of size k, each element must appear equally often. Throughout, binomial coefficients with negative lower indices are interpreted as zero.

For two distinct elements i,j, the following computation counts all blocks according to the joint multiplicities of i and j, summing the products of their multiplicities over all blocks yields the weighted pairwise concurrence (a Vandermonde-like convolution):

λ=∑s=1k∑t=1k−sstv+k−s−t−3k−s−t=v+k−1k−2,

as derived in detail in Appendix B, using repeated hockey-stick identities; see also Gould [14] for related multiset summations. Thus D(v:k) is balanced. □

### 2.4 Illustrative Examples

To demonstrate the construction of the master design D(v:k), we examine the compositions and design parameters for specific values of v and k.

#### 2.4.1 Case 1: v=3,k=2 (balanced 3-ary design)

For block size k=2, the design is a (k+1)=3-ary design. The design parameters are:

b=3+2−12=6,r=41=4,λ=40=1

The blocks are categorized by their multiplicity patterns in Table 1.

This construction yields a (v,b,r,k,λ)=(3,6,4,2,1) design, coinciding with the complete ternary multiset design on three treatments.

#### 2.4.2 Case 2: v=3,k=3 (balanced 4-ary design)

For block size k=3, the design is a (k+1)=4-ary design. The design parameters are:

b=3+3−13=10,r=52=10,λ=51=5

The block structure becomes more complex, as shown in Table 2.

#### 2.4.3 Case 3: v=2,k=3 explicit λ calculation)

To address the precision of the pairwise index λ during block deletion, we examine the smallest non-trivial case D(2:3). The master design contains b=2+3−13=4 blocks:

$$B_{1}\;:\;\left\{1^{3}\right\},B_{2}\;:\;\left\{2^{3}\right\},\;B_{3}\;:\;\left\{1^{2},2\right\},B_{4}\;:\;\left\{1,2^{2}\right\}$$

The master pairwise index is λmaster=41=4. Verifying manually via the sum of products:

λ=(3⋅0)+(0⋅3)+(2⋅1)+(1⋅2)=4.

Applying the deletion rule for s=2 (deleting blocks where $x_{i}=3$), we remove $B_{1}$ and $B_{2}$. The resulting ternary design $D_{\mathrm{sub}}=\left\{\left\{1^{2},2\right\},\left\{1,2^{2}\right\}\right\}$ has:

b=2,r=3,λ=(2⋅1)+(1⋅2)=4.

Here “ternary“ refers to the cardinality of the multiplicity set {0, 1,2}, with n=3, rather than to the number of distinct treatments per block; this convention is used throughout Section 3. The example is intended purely to illustrate preservation of the pairwise index under deletion in the smallest setting with multiplicities exceeding one and, from a classical block-design perspective, should be viewed as essentially trivial.

### 2.5 Relation to multiset designs

In comparison to Assaf et al. [11], whose ${MB}_{t}(v,k,\lambda )$ framework allows general t-balance with multiplicity counting and permits repeated/non-distinct blocks, our D(v:k) realizes a specific ${MB}_{2}(v,k,\lambda )$ using all *distinct* multisets of fixed size k. This enforces block distinctness by construction (unlike their general multiset designs with possible repetitions or variable sizes), while preserving $S_{v}$-symmetry for systematic deletions and yielding explicit pairwise parameters (Theorem 2.1) with arity families absent in prior work-prioritizing statistical applications like variance control via λ.

## 3 Symmetry-Preserving Truncations

A central feature of the master design D(v:k) is its complete symmetry with respect to the v elements. As a consequence, subdesigns obtained by deleting blocks according to multiplicity constraints inherit balance properties, provided the deletion rule is applied uniformly across elements.

For s=0, 1,…,k, let N(s) denote the number of blocks in D(v:k) in which a fixed element occurs exactly s times. By symmetry, N(s) is independent of the choice of element and is given by

N(s)=v+k−s−2k−s.

This follows by fixing $x_{i}=s$ and distributing k−s units over the remaining v−1 elements by stars-and-bars, giving (v−1)+(k−s)−1k−s=v+k−s−2k−s blocks. Throughout this section, balance preservation is established by exploiting the invariance of the deletion rules under permutations of the v element labels.

**Proposition 3.1.** *Let* D(v:k) *denote the master multiset design consisting of all distinct multisets of size* k from a v-*set. Fix an integer* s *with* 0≤s≤k−1, *and consider the deletion rule that removes all blocks in which some element occurs with multiplicity exceeding* s.

*If* s<(k+1)/2, *pairwise balance need not be preserved whenever there exist blocks in* D(v:k) *containing two distinct elements each with multiplicity exceeding* s. *Conversely, if* s≥(k+1)/2, *then every deleted block contains exactly one dominant element (i.e., an element* d *with* $x_{d}>s$*), and the deletion preserves pairwise balance by* $S_{v}$-*invariance*.

*Proof*. We consider the two regimes separately.

**Case 1:** s<(k+1)/2. Assume

$$s<\frac{k+1}{2}.$$

Then 2(s+1)≤k, and hence there exist blocks in D(v:k) of the form

$$B=\left\{i^{s+1},j^{s+1},\;\text{remaining elements summing to}\;k-2(s+1)\right\},$$

where i≠j. In particular, when k=2(s+1), the block

$$B=\left\{i^{s+1},\;j^{s+1}\right\}$$

exists.

Such a block contains two elements whose multiplicities exceed s and is therefore removed by the deletion rule. Fix an unordered pair {i,j}. The deleted block B contributes

$$x_{i}x_{j}=(s+1{)}^{2}>0$$

to the pairwise index λ(i,j). By contrast, for any other unordered pair {i,ℓ} with ℓ≠j, the contribution of B is

$$x_{i}x_{\ell }=(s+1)\cdot 0=0.$$

Thus, deletion of B removes a positive contribution from the pair {i,j} but removes no contribution from many other pairs. Consequently, the total reduction in λ(i,j) differs from that of other unordered pairs, and pairwise balance is destroyed.

**Case 2:** s≥(k+1)/2. Assume

$$s\geq \frac{k+1}{2}.$$

Suppose, for contradiction, that a block contains two distinct elements i≠j such that

$$x_{i}>s\;\text{and}\;x_{j}>s.$$

Then

$$x_{i}+x_{j}\geq (s+1)+(s+1)=2s+2.$$

Since s≥(k+1)/2, it follows that 2s+2≥k+1>k, which contradicts the block-size constraint

$$\sum_{r=1}^{v}x_{r}=k.$$

Hence, no block in D(v:k) can contain more than one element with multiplicity exceeding s.

Therefore, every deleted block contains exactly one dominant element d with $x_{d}>s$.

### Preservation of pairwise balance.

When each deleted block has exactly one dominant element, any deleted block contributes nonzero concurrence only to unordered pairs involving that dominant element. Since the deletion rule depends solely on multiplicity patterns and is invariant under permutations of the v element labels, the collection of deleted blocks is invariant under the action of the symmetric group $S_{v}$. Consequently, each unordered pair of elements {i,j} loses the same total contribution to the pairwise index, and pairwise balance is preserved; in later sections, the uniqueness of the dominant element under s≥(k+1)/2 is used to obtain explicit formulas for the pairwise index adjustments.

*Remark* 3.2. Proposition 3.1 provides a *sufficient* condition for balance preservation. For certain small (k,s) (e.g., k=4,s=2), balance may still hold, but (k+1)/2 provides a uniform, dimension-free threshold.

### 3.1 Deletion by maximum multiplicity

For a fixed integer m with 1≤m≤k, define $D^{\left(m\right)}$ to be the subdesign obtained from D(v:k) by deleting all blocks in which any element occurs with multiplicity at least m.

**Lemma 3.3.** *Any subdesign obtained by applying a multiplicity-based deletion rule that is invariant under permutations of the element labels is pairwise balanced.*

In particular, such invariant rules remove the same total contribution from every unordered pair of distinct elements, thereby preserving constant pairwise concurrence.

*Proof*. Let E={1,…,v} denote the treatment set and $G=S_{v}$ the symmetric group acting on E by permutation. This action extends naturally to the block set by permuting element labels within each block.

Let ${ℬ}_{d}$ denote the collection of deleted blocks. By construction, the deletion rule depends only on the multiplicity pattern within a block and not on the specific labels of the elements. Hence for every g∈G,

$$g{ℬ}_{d}={ℬ}_{d},$$

so ${ℬ}_{d}$ is invariant under the action of G.

Fix two distinct elements i,j∈E and define

$$L(i,j)=\sum_{B\in {ℬ}_{d}}x_{iB}x_{jB},$$

the total concurrence contribution of the deleted blocks to the unordered pair {i,j}.

Since G acts transitively on the set of unordered pairs

𝒫={{a,b}:a≠b},

for any other unordered pair $\left\{i^{\prime },j^{\prime }\right\}\in 𝒫$ there exists g∈G such that

$$g(\{i,j\})=\left\{i^{\prime },j^{\prime }\right\}.$$

Using invariance of ${ℬ}_{d}$,

$$L\left(i^{\prime },j^{\prime }\right)=\sum_{B\in {ℬ}_{d}}x_{i^{\prime }B}x_{j^{\prime }B}=\sum_{B\in {ℬ}_{d}}x_{g(i)B}x_{g(j)B}=\sum_{B\in {ℬ}_{d}}x_{i,g^{-1}B}x_{j,g^{-1}B}.$$

Since $g^{-1}{ℬ}_{d}={ℬ}_{d}$, reindexing gives

$$L\left(i^{\prime },j^{\prime }\right)=\sum_{B\in {ℬ}_{d}}x_{iB}x_{jB}=L(i,j).$$

Thus every unordered pair loses the same total concurrence contribution from the deleted blocks.

Let $\lambda_{\text{master}}$ denote the pairwise index of the master design. The resulting design therefore has pairwise index

$$\lambda =\lambda_{master}-L(i,j),$$

which is independent of the chosen pair {i,j}. In the constructions of Section 3 (e.g., Theorem 3.8), the additional condition

s≥(k+1)/2

(Proposition 3.1) ensures each deleted block has a unique dominant element, further simplifying the pairwise adjustment while maintaining this invariance. Hence the reduced design is pairwise balanced. **This argument requires only** $S_{v}$-**invariance of the set of deleted blocks and does not assume uniqueness of any dominant element.**

The deletion rules employed here are defined purely in terms of maximum allowable multiplicities and are therefore independent of treatment labels, making them invariant under the $S_{v}$-action. By Lemma 3.3, this invariance ensures that all derived subdesigns inherit pairwise balance from the master design D(v:k). Moreover, the complete symmetry of the incidence structure preserves the information matrix in the completely symmetric form

$$C=\rho \left(I_{v}-\frac{1}{v}J_{v}\right)$$

for some ρ>0 determined by the adjusted pairwise index λ. This structure is the standard prerequisite for universal optimality [17; 18]. Consequently, this provides the theoretical bridge between the multiset framework established in Section 2 and the equireplicated, variance-balanced n-ary families constructed in the subsequent sections.

### 3.2 Balanced *k*-ary and (*k*−1)-ary designs

We first consider the simplest deletion schemes.

**Theorem 3.4.** *For any* v≥k≥2, *deleting the* v *blocks in which a single element appears with multiplicity* k *yields a balanced* k-*ary design with*

b=v+k−1k−v.

Since the deleted blocks are invariant under permutations of the element labels, the pairwise index of the resulting design remains unchanged. This represents the largest reduction in arity while maintaining the master pairwise index obtainable via deletion of pure blocks (those with a single treatment repeated k times) without altering the pairwise index.

*Proof*. There is exactly one block of the form {i,i,…,i} for each i∈E. Deleting these v blocks enforces a maximum multiplicity of k−1 in every remaining block. The deleted blocks are characterized solely by their multiplicity pattern (k) and are invariant under permutations of the v element labels; consequently, the induced action of $S_{v}$ removes the same total contribution from every unordered pair of distinct elements. By Lemma 3.3, the resulting design is balanced. Deleted pure blocks contribute zero to the pairwise index λ (single element), so

λsub=λmaster=v+k−1k−2

□

**Theorem 3.5** (Balanced (k−1)-ary designs). For v>k≥3, *deleting all blocks in which any element occurs with multiplicity* k *or* k−1 *yields a balanced* (k−1)-*ary design with parameters:*

b=v+k−1k−v−v(v−1),r=kbv,

and a closed-form expression for the resulting pairwise index:

λ=v+k−1k−2−2(k−1).

*Proof*. The number of blocks in the master design D(v:k) is bmaster=v+k−1k. We identify two types of blocks to be deleted based on multiplicity patterns:

- Blocks of type (k,0,…,0): There are exactly v such blocks (e.g., {i,i,…,i} for each i∈E)
- Blocks of type (k−1,1,0,…,0): For each ordered pair of distinct elements (i,j), there is exactly one block where element i occurs k−1 times and element j occurs once. There are v(v−1) such blocks.

Summing these gives the total deleted blocks, $v+v(v-1)=v^{2}$, leading to the stated expression for b. Assuming v>k ensures b>0 and a non-degenerate design. The replication number r follows from the standard identity bk=vr.

To determine λ, consider an unordered pair of distinct elements {i,j}. In the master design, the pairwise index is λmaster=v+k−1k−2.

- Type (i) blocks contribute k×0=0 to the concurrence of any pair.
- Type (ii) blocks: The pair {i,j} appears together with non-zero multiplicity only in the blocks $\left\{i^{k-1},j^{1}\right\}$ and $\left\{j^{k-1},i^{1}\right\}$. The contribution from the first is (k−1)×1 and from the second is 1×(k−1).

The total contribution removed from the pairwise index for any pair {i,j} is thus (k−1)+(k−1)=2(k−1). Since the master design is $S_{v}$-symmetric and the deletion rule is label-invariant, every unordered pair {i,j} is contained in exactly two type-(k−1,1) blocks (one with i dominant and j single, one symmetric). Thus the reduction of 2(k−1) is uniform across all pairs. By Lemma 3.3, the design remains balanced.

This uniform reduction of 2(k−1) across all pairs arising from the symmetric deletion of type-(k−1, 1) blocks ensures that the resulting design remains balanced, with pairwise index

$$\lambda =\lambda_{\mathrm{master}}-2(k-1).$$

Consequently, the design inherits the symmetry-based optimality of the master design through a completely symmetric information matrix

$$C=\rho \left(I_{v}-\frac{1}{v}J_{v}\right)$$

□

*Example 3.6* (Contrast with classical designs). Let v=4 and k=3. The master design D(4:3) has b=63=20 blocks and λmaster=61=6. We construct a balanced (k−1)=2 ary (binary) design by deleting:

- v=4 blocks of type {i,i,i}.
- v(v−1)=12 blocks of type {i,i,j}.

The resulting design has

$$b=20-4-12=4,\;r=\frac{3\times 4}{4}=3,\;\lambda =6-2(3-1)=2.$$

This yields a balanced binary design with parameters (v,b,r,k,λ)=(4,4,3,2,2). Unlike a classical BIBD or the affine plane of order 2, which has λ=1 and b=6 blocks, the present design exhibits higher pairwise concurrence with fewer blocks. This example illustrates that balanced n-ary designs obtained via systematic block deletion need not coincide with classical designs, even when reduced to the binary case.

### 3.3 Balanced (s+1)-ary designs

More generally, let s satisfy (k+1)/2≤s≤k−1, and let $D_{s}$ denote the subdesign consisting of all blocks in which no element occurs more than s times.

*Remark* 3.7 (When does $S_{2}$ vanish?). In Appendix C, the term $S_{2}$ accounts for deleted blocks whose dominant element is neither i nor j. Such blocks contribute to the pairwise concurrence {i,j} only if both i and j appear with positive multiplicity, which requires

k−t≥2,

where t is the dominant multiplicity.

Since t≥s+1 for all deleted blocks, this condition is equivalent to

k−(s+1)≥2⟺s≤k−3.

Consequently,

$$S_{2}=0\;\text{if and only if}\;s\geq k-2.$$

**Theorem 3.8** (Balanced (s+1)-ary designs). *Let* v≥k≥2 *and* s≥(k+1)/2 *(the regime in which pairwise balance is preserved by Proposition 3.1). Let* $D_{s}$ *denote the subdesign obtained from the balanced* (k+1)-*ary master design* D(v:k) *by deleting all blocks in which any element occurs more than* s *times. Then* $D_{s}$ *is a balanced* (s+1)-*ary design with*

b=v+k−1k−vv+k−s−2k−s−1,r=kbv,

and pairwise index

λ=v+k−1k−2−2S1+(v−2)S2,

*where* $S_{1}$ *and* $S_{2}$ *are defined in* Appendix D and Appendix C *(with the general triple-sum expression for* $S_{2}$).

*If in addition* s≥k−2 *(maximum remaining mass* k−(s+1)≤1*), then* $S_{2}=0$ *and the pairwise index simplifies to*

λ=v+k−1k−2−2⋅k+sv+v−1v+1v+k−s−2k−s−2.

*Proof*. Fix an element i∈E. We first determine how many blocks of the master design D(v:k) are deleted because element i appears with multiplicity exceeding s.

For a fixed multiplicity t>s, the remaining k−t units are distributed among the other v−1 elements. The number of such blocks is therefore

(v−1)+(k−t)−1k−t=v+k−t−2k−t.

Summing over all admissible values of t gives

∑t=s+1kv+k−t−2k−t.

By the hockey-stick identity (Appendix D),

∑t=s+1kv+k−t−2k−t=v+k−s−2k−s−1.

This quantity is independent of the choice of i. Hence the total number of deleted blocks is

vv+k−s−2k−s−1.

The above identity holds for k−s−2≥0. If k−s−2<0 (i.e., s≥k−1), the sum is empty and equals zero by convention. Hence, the number of remaining blocks is

b=v+k−1k−vv+k−s−2k−s−1.

Replication follows from bk=vr :

$$r=\frac{kb}{v}.$$

#### Balance.

The deletion rule depends only on multiplicity patterns and not on element labels. Therefore the collection of deleted blocks is invariant under the symmetric group $S_{v}$. By Lemma 3.3, this invariance implies that every unordered pair {i,j} loses the same total contribution. Hence the remaining design is pairwise balanced.

#### Pairwise index.

Let λmaster=v+k−1k−2. The reduction in the pairwise index due to the deleted blocks is $\lambda_{𝒟}=2S_{1}+(v-2)S_{2}$, where $S_{1}$ (Appendix D) and $S_{2}$ (Appendix C) are the adjustment terms for blocks in which one of i or j is dominant $\left(S_{1}\right)$ or neither is dominant $\left(S_{2}\right)$.

Under the condition s≥(k+1)/2, each deleted block has a unique dominant element (Proposition 3.1). For $S_{2}>0$, there must exist a deleted block with dominant d∉{i,j} and non-zero concurrence contribution $x_{i}x_{j}>0$, which requires $x_{i}\geq 1$ and $x_{j}\geq 1$ (hence $x_{i}+x_{j}\geq 2$). However, in any deleted block the dominant multiplicity satisfies $x_{d}\geq s+1$, so

$$x_{i}+x_{j}\leq k-x_{d}\leq k-(s+1).$$

Thus $S_{2}>0$ only if k−(s+1)≥2, i.e., s≤k−3. Equivalently, $S_{2}=0$ whenever s≥k−2. In this regime, the adjustment simplifies to $\lambda_{𝒟}=2S_{1}$, and

λ=λmaster−2S1=v+k−1k−2−2⋅k+sv+v−1v+1v+k−s−2k−s−2.

Equivalently, $S_{2}=0$ whenever s≥k−2. In this regime, the adjustment simplifies to $2S_{1}$. Direct enumeration for small (v,k,s) confirms the formula; for instance, with v=4,k=4, s=2, we obtain λ=15. □

*Remark* 3.9 (Balance and symmetry). The subdesign $D_{s}$ is balanced because the deletion rule depends only on multiplicity patterns and is invariant under permutations of the v element labels. By Lemma 3.3, the total contribution removed from each unordered pair {i,j} is identical, preserving the constant pairwise index. Consequently, $D_{s}$ inherits the $S_{v}$-symmetry and optimality properties of the master design D(v:k).

*Remark* 3.10 (Pairwise index simplification). In Theorem 3.8, the general expression for the pairwise index is

λ=v+k−1k−2−2S1+(v−2)S2,

where $S_{1}$ and $S_{2}$ are defined in Appendix D and Appendix C, respectively.

Under the condition s≥k−2, no deleted block contains both elements i and j unless one is the dominant element, so $S_{2}=0$, and the pairwise index simplifies to

λ=v+k−1k−2−2S1.

For $(k+1)/2\leq s<k-2,S_{2}$ may be positive, and the full expression must be used.

*Remark* 3.11 (Special cases). If s=k−1, then no blocks are deleted, $D_{s}$ coincides with the master (k+1)-ary design D(v:k), and λ=λmaster=v+k−1k−2. Similarly, for s=k, the design remains unchanged.

*Remark* 3.12 (Generalization and optimality). Theorem 3.8 provides a unified framework for constructing balanced (s+1)-ary designs from the master design. The systematic, symmetrypreserving deletions allow explicit control of replication and pairwise indices, making these designs directly applicable to statistical experiments requiring equireplication and minimal contrast variances.

**Note:** Theorem 3.8 is stated under the weaker condition s≥(k+1)/2, which ensures each deleted block has a *unique* dominant element. However, the general expressions for the design parameters rely on the adjustment terms $S_{1}$ and $S_{2}$, which are defined in Appendix D and Appendix C, respectively. Specifically, $S_{1}$ is given in closed form in Appendix D, while $S_{2}$ is defined by the triple sum derived in Appendix C. Under the condition s≥k−2, the contribution $S_{2}$ vanishes ($S_{2}=0$), yielding the simplified fractional form for the pairwise index λ. The condition s≥(k+1)/2 is used only to guarantee that each deleted block has a unique dominant element; pairwise balance still follows from $S_{v}$-invariance as in Lemma 3.3.

*Remark* 3.13. The binomial identity ∑t=s+1kv+k−t−2k−t=v+k−s−2k−s−1 is valid for k−s−1≥0 (i.e., s≤k−1). When k−s−1<0 (equivalently s>k−1), the summation index set is empty, and by convention the sum equals zero. In this regime no blocks are deleted, so $D_{s}$ coincides with the master design and $\lambda =\lambda_{\text{master}}$.

##### Numerical examples and verification

In this section, we provide two numerical examples to demonstrate the construction of truncated balanced n-ary designs from master designs and verify the parameter formulas derived in Theorem 3.8.

*Example* 3.14. Let v=4 and k=4. We consider the balanced (k+1)-ary master design D(4:4). We construct the truncated design $D_{s}$ by setting the maximum multiplicity s=2. Blocks with multiplicity patterns (3, 1) or (4, 0) are removed.

1. **Explicit block counts** (*b*): For each fixed i∈{1, 2,3, 4}, there are exactly 3 blocks of the form {i,i,i,j},j≠i. Indeed, once i is chosen, the remaining element j can be any of the other three symbols. Therefore, the total number of such blocks is 4×3=12. Additionally, there are 4 blocks of the form {i,i,i,i}, which are also deleted. Hence, a total of 16 blocks are removed. The master design size is 4+4−14=35. Thus, b=35−16=19.
2. **Replication Number** (r): $r=\frac{kb}{v}=\frac{4\times 19}{4}=19$.
3. **Pairwise Index** (λ): Using the derived formula with $S_{1}=3$ (the number of deleted blocks in which exactly one of the two elements is dominant), we have λ=4+4−12−2S1=21−6=15.

The construction yields a balanced ternary design with parameters (4, 19, 19, 4, 15). Note that although r=b here, this is accidental for (v,k,s)=(4, 4,2) and does not hold generally.

*Example* 3.15 (Case (v,k,s)=(5,4,2)). Let v=5 and k=4. We consider the balanced (k+1)-ary master design D(5:4), consisting of all possible multisets of size 4 . We construct the truncated design $D_{2}$ by imposing a maximum multiplicity threshold of s=2. This process removes all blocks where any treatment appears with multiplicity $x_{i}>2$ (specifically, blocks with multiplicity patterns (4) and (3, 1)).

- **Block count** (*b*): The initial master design contains
b0=v+k−1k=5+4−14=84=70blocks.
  The deleted blocks consist of:
  - *Type (4):* Pure blocks where one element appears four times. There are v=5 such blocks.
  - *Type (3,1)*: Blocks where one element appears three times and another appears once. There are v(v−1)=5×4=20 such blocks.
  The total number of deleted blocks is 5+20=25. Thus, the truncated design has
b=70−25=45blocks.
- **Replication number** (r): By the property of balance and the identity bk=vr, the replication number is
$$r=\frac{bk}{v}=\frac{45\times 4}{5}=36.$$
- **Pairwise index** (λ): We verify the index λ using two distinct methods to ensure consistency with Theorem 3.8.

*Method 1 (Enumeration by Block Type):* For a fixed pair of treatments {i,j}, we sum the concurrence $x_{i}x_{j}$ across all kept block types:

- **Type (2,2):** One block {i,i,j,j} contributes 2×2=4.
- **Type (2,1,1):**
  - Blocks {i,i,j,a} for a≠i,j (3 blocks): 3×(2×1)=6.
  - Blocks {j,j,i,a} for a≠i,j (3 blocks): 3×(2×1)=6.
  - Blocks {a,a,i,j} for a≠i,j (3 blocks): 3×(1×1)=3.
- **Type (1,1,1,1):** Blocks containing {i,j} and two other distinct elements. There are v−2k−2=32=3 such blocks, each contributing 1×1=3.

Total λ=4+6+6+3+3=22.

*Method 2 (Formula Verification):* According to Theorem 3.8, the index is λ=v+k−1k−2−2S1. For (v,k,s)=(5,4,2), the term $2S_{1}$ represents the total concurrence lost from deleted blocks where one element of the pair is dominant ($x_{i}=3$). For pair {1,2}, the deleted block {1,1,1,2} contributes 3×1=3, and {2,2,2,1} contributes 1×3=3. Thus,

λ=82−(3+3)=28−6=22.

Both the combinatorial enumeration of kept block types and the application of the general index formula from Theorem 3.8 yield identical results. This confirms that the proposed truncation of the complete multiset space successfully produces a balanced ternary design with parameters:

(v,b,r,k,λ)=(5,45,36,4,22).

These examples illustrate the consistency between direct enumeration of concurrence contributions and the general formulas from Theorem 3.8, validating the adjustment terms $S_{1}$ and $S_{2}=0$.

### 3.4 Complementary designs

The deleted blocks in Theorem 3.8 themselves form a balanced design.

*Remark* 3.16. The condition s≥(k+1)/2 is sufficient to ensure no block contains more than one element with multiplicity exceeding s, which is required for the symmetry-based deletion argument in Lemma 3.3 to preserve pairwise balance without additional adjustments.

While this condition is not strictly necessary in all parameter regimes-for example, when k=4 and s=2 (where s<2.5 but no blocks with two elements exceeding multiplicity 2 exist)-it provides a general, dimension-independent guarantee that simplifies the balance preservation argument. If s<(k+1)/2 and blocks with multiple high-multiplicity elements exist (for example, k=6,s=2, block {i,i,i,j,j,j}), then deletion removes unequal contributions from different unordered pairs and pairwise balance generally fails.

Throughout this subsection, we assume s≥(k+1)/2 unless explicitly stated otherwise.

**Theorem 3.17** (Complementary Design). Let (k+1)/2≤s≤k−1 (*cf. Proposition 3.1*). *The collection of blocks deleted in Theorem 3.8 forms a balanced* (k+1)-*ary design*, distinct from the master design in all non-degenerate cases D(v:k) *(except in degenerate cases), in which each block contains exactly one element with multiplicity greater than s (the dominant element) and the remaining elements have multiplicity at most* s. *The design parameters are*

b=vv+k−s−2k−s−1,r=kbv,

and pairwise index

$$\lambda =2S_{1}+(v-2)S_{2},$$

*where* $S_{1}$ *and* $S_{2}$ *are defined in* Appendix D *and* Appendix C, *respectively. When* s≥k−2 *(maximum remaining mass* ≤1), $S_{2}=0$ *and the pairwise index simplifies to* $\lambda =2S_{1}$.

*Proof*. By construction, each deleted block contains exactly one dominant element d with multiplicity >s (Proposition 3.1). The remaining $k-x_{d}$ units are distributed among the other v−1 elements with multiplicity at most s.

The deletion rule depends only on multiplicity patterns and is invariant under permutations of the v elements. By Lemma 3.3, the total pairwise contribution from the deleted blocks is uniform across all unordered pairs {i,j}. Hence, the deleted blocks themselves form a balanced design.

The number of blocks with a given dominant element is

v+k−s−2k−s−1,

so summing over all v elements gives the total block count b=vv+k−s−2k−s−1. (For s=k−1, the binomial is zero by convention, yielding b=0, the empty design.) The replication number follows from bk=vr, yielding r=kb/v.

The pairwise index is determined by the contributions of the dominant and non-dominant elements as in Appendix C, giving $\lambda =2S_{1}+(v-2)S_{2}$. When s≥k−2, the maximum remaining mass k−(s+1)≤1, so no deleted block can contain both i and j with positive multiplicity when the dominant is neither; thus $S_{2}=0$ and $\lambda =2S_{1}$.

*Remark* 3.18 (Statistical interpretation). The complementary design captures the blocks removed to enforce the ( s+1)-ary constraint in $D_{s}$. It retains complete $S_{v}$-symmetry and pairwise balance, and, as a consequence, its information matrix under the fixed-effects model is completely symmetric. This allows explicit computation of contrast variances, providing a structured design family complementary to $D_{s}$ for experimental applications.

*Remark* 3.19 (Special cases). If s=k−2, the complementary design contains exactly those blocks in which a single element has multiplicity k−1 and all others have multiplicity ≤k−2. For s=k−1, no blocks are deleted, so the complementary design is empty.

*Example* 3.20. Let v=4,k=4, and s=2. The master design D(4:4) has parameters

bm=4+4−14=35,λm=4+4−12=21.

Applying Theorem 3.8 with s=2 deletes all blocks containing an element with multiplicity at least 3. The number of deleted blocks is vv+k−s−2k−s−1=441=16. This yields a ternary (s+1=3)-ary subdesign $D_{2}$ with b=35−16=19 blocks. Using the formula in Theorem 3.8, the pairwise index is:

λ=21−2⋅4+(2)(4)+4−14+14+4−2−24−2−2=21−2⋅15540=21−6=15.

*Example* 3.21. Continuing from Example 3.20 with v=4,k=4, and s=2, the deleted 16 blocks are of the form {i,i,i,j} for i≠j. These blocks form a balanced k+1=5-ary design. According to the formulas in Theorem 3.17, the design parameters are b=16 and r=16. The pairwise index is:

λ=2⋅4+(2)(4)+4−14+14+4−2−24−2−2=2⋅15540=6,

since each pair {i,j} receives a contribution of 3×1=3 from the block {i,i,i,j} and 1×3=3 from the block {j,j,j,i}.

The parameters of the master design and its derived families are summarized in Table 3

Table 4 confirms numerically, for a representative small case, the consistency of the general formulas summarized in Table 3 and illustrates how balance is preserved under successive arity-reducing deletions.

## 4 Discussion and Further Directions

The constructions presented in this paper establish a symmetric combinatorial framework for balanced n-ary designs. The master design D(v:k) is invariant under the symmetric group $S_{v}$, and this invariance is preserved across all derived subdesigns obtained via uniform, label-invariant block deletions. These properties mirror those of classical optimal designs, in the sense of complete symmetry of the information matrix for main-effects models; correspondingly, all derived families satisfy natural analogues of Fisher’s inequality b≥v (Preece [12]).

While our focus remains combinatorial, the framework is directly relevant to practical design considerations. The structured deletion mechanisms generate subdesigns with varying multiplicities, providing a class of candidate blocks for resampling schemes, dose-response experiments, and clinical trials. For instance, balanced n-ary designs apply to crossover experiments in which residual treatment effects must be balanced to minimize carryover bias Aggarwal and Jha [16]. In agriculture, they evaluate general combining ability in diallel crosses Shah and Dey [4], while weighing designs leverage ternary variants for precise measurement with minimal trials. Under a fixed-effects model, the variance of contrast estimators is inversely proportional to λ, enabling precise control via systematic deletions Pukelsheim [18, Ch. 9].

The current framework is combinatorially distinct from Efficiency Balanced Sample Designs (EBSD) Srivastav and Srivastav [13], which focus on inferential efficiency. However, the structured design space defined by the master and derived n-ary designs provides a natural foundation for exploring inferentially motivated criteria, such as Monte Carlo power and Type-I error rates under non-normality, in future work.

### 4.1 Statistical Efficiency and Empirical Variance

Under the additive fixed-effects model in which each entry in a multiset block corresponds to an independent observation receiving the corresponding treatment (with independent errors across all bk observations), the information matrix for the treatment effects is diagonal with entries equal to the replication numbers $r_{i}$. This corresponds to a completely randomized design without block effects; in Section 4.2 we return to the usual intra-block information matrix C when block effects are included. We emphasize that this model treats each unit within a block as an independent observation receiving the corresponding treatment. Block effects are ignored here for analytic simplicity; extensions to mixed block models are discussed below. For the equireplicated designs constructed herein, $r_{i}=r=kb/v$ for all i, with closed-form expressions provided for b and hence r. In this model, the variance of any elementary treatment contrast estimator ${\overset{ˆ}{\tau }}_{i}-{\overset{ˆ}{\tau }}_{j}$ is

$$Var\left({\overset{ˆ}{\tau }}_{i}-{\overset{ˆ}{\tau }}_{j}\right)=\frac{2\sigma^{2}}{r}.$$

The $S_{v}$-symmetry of the constructions ensures equireplication, yielding uniform precision across all contrasts.

To illustrate this efficiency gain, consider the (k−1)-ary design with v=4,k=3, and resulting parameters b=4,r=3. The theoretical contrast variance is $2\sigma^{2}/3\approx 0.667\sigma^{2}$. A simulation study comprising 1000 replications confirms the advantage of equireplication: randomly generated multiset arrangements (allowing repeats within blocks) typically exhibit unequal replication numbers across treatments, resulting in higher average contrast variances (approximately $0.856\sigma^{2}$ in a representative run). This corresponds to roughly a 22% increase in variance relative to the equireplicated case, providing empirical support for the benefit of systematic constructions that maintain constant replication. Detailed R code for this simulation is provided in Appendix E.

Note that in alternative models incorporating fixed or random block effects (common in repeated-measure or crossover experiments), the high pairwise concurrence λ achieved by the proposed designs would additionally contribute to balanced intra-block information and further reduced contrast variances, though closed-form expressions under such models are more involved.

### 4.2 Optimality considerations

Due to the complete $S_{v}$-symmetry of the master design D(v:k) and the symmetry-preserving nature of the deletion schemes, all derived balanced n-ary designs possess a completely symmetric intra-block information matrix under the standard fixed-effects linear model with fixed block effects and equal block sizes. Specifically, the information matrix for treatment effects takes the form

$$C=\rho \left(I_{v}-\frac{1}{v}J_{v}\right),$$

where $\rho =\frac{\lambda v}{k}$. This structure follows from the constant off-diagonal entries −λ/k in the normalized concurrence contribution and the uniform diagonal adjustment ensured by symmetry.

#### Connection to classical optimality theory:

The above form coincides with the information matrix of universally optimal designs in the sense of Kiefer [17]. In particular, the proportional-to-identity structure on the contrast space implies optimality under all concave, permutation-invariant optimality criteria, including *A*, *D*-, *E*-optimality. This follows directly from Kiefer’s equivalence theorem and the general theory of optimal experimental design (Pukelsheim [18], Chapter 6). Hence, the proposed designs are universally optimal within the class of approximate designs under the fixed-effects model.

The matrix C has eigenvalue ρ with multiplicity v−1 (on the contrast space) and eigenvalue 0 with multiplicity 1 (for the all-ones vector). The A-optimality criterion, which minimizes the average variance of the best linear unbiased estimators of all elementary treatment contrasts, is equivalent to minimizing the trace of the pseudoinverse $C^{-}$ over the contrast subspace:

$$trace\left(C^{-}\right)=\frac{v-1}{\rho }.$$

The average variance of a pairwise contrast is thus $2\sigma^{2}/\rho =2k\sigma^{2}/(\lambda v)$. For fixed v and k (and hence fixed total number of observations bk in comparisons across families), larger values of the pairwise index λ increase ρ, reducing average contrast variances and improving A-efficiency among equireplicated variance-balanced designs.

Compared to classical binary balanced incomplete block designs (where within-block repetition is prohibited), the present n-ary constructions can achieve substantially larger λ through controlled repetitions, leading to lower contrast variances in applications where repeated treatments within blocks are meaningful (e.g., dose-response or repeated-measure experiments). Formal efficiency comparisons relative to exact universally optimal designs for specific parameter settings are left for future investigation.

*Remark* 4.1 (Relation to EBSD). The present constructions are complementary in spirit to the Efficiency Balanced Sample Design (EBSD) framework of Srivastav and Srivastav [13]. While EBSD(n) focuses on optimizing resampling efficiency under explicit variance criteria, our results provide a structured combinatorial design space with controlled multiplicities and explicit balance parameters. In this sense, the proposed families may be viewed as furnishing candidate design classes within which inferentially motivated criteria, such as those underlying EBSD(n), can be systematically explored in future work.

### 4.3 Fisher-Type Bounds for n-Ary Families

Fisher’s inequality, b≥v, originally established for BIBDs, extends to n-ary equireplicated designs with constant weighted pairwise concurrence (Preece [12]).

More generally, Fisher-type lower bounds for connected n-ary designs follow from rank properties of the incidence matrix N. For any connected design, the information matrix

$$C=R-\frac{1}{k}NN^{⊤}$$

satisfies rank(C)=v−1. Since

rank(C)≤rank(N)≤b,

it follows that

b≥v−1.

This is the fundamental Fisher bound for connected multiset designs.

If, in addition, the design contains no repeated blocks and is non-degenerate (i.e., with k<v (treatments not nested)), then equality b=v−1 cannot occur (Preece [12]), and the bound strengthens to

b≥v.

**Theorem 4.2.** *Every connected equireplicated* n-*ary design satisfies* b≥v−1. *If the design has no repeated blocks, then* b≥v, *with equality* b=v *corresponding to the symmetric case in which the number of blocks equals the number of treatments.*

*Proof*. For a connected design, rank(C)=v−1. Since $C=R-\frac{1}{k}NN^{⊤}$,

rank(C)≤rank(N)≤b,

which yields b≥v−1.

If no block is repeated, the case b=v−1 is impossible (Preece [12]); hence b≥v. When equality holds, the design is symmetric.

See John and Williams 19 and Dey 20 for detailed rank-based arguments in generalized block-design settings.

We verify these bounds for the following families:

- **Master design:** b=v+k−1k>v for all k≥2.
- (k−1)-**ary design:** b=v+k−1k−v2≥v for v>k≥3.
- (s+1)-**ary design:** b≥v holds whenever s≥(k+1)/2.

## Figures and Tables

**Table 1:** Block configurations for *v* = 3, *k* = 2.

Pattern	Specific Blocks	Count
Single Multiplicity (*x_i_* = 2)	{1, 1}, {2, 2}, {3, 3}	3
Distinct Pairs (*x_i_* = 1, *x_j_* = 1)	{1, 2}, {1, 3}, {2, 3}	3

**Table 2:** Compact block configurations for *v* = 3, *k* = 3.

Pattern	Specific Blocks	Count
Pure (*x_i_* = 3)	{1^3^}, {2^3^}, {3^3^}	3
Mixed (*x_i_* = 2, *x_j_* = 1)	{1^2^, 2}, {1^2^, 3}, {2^2^, 1}, {2^2^, 3}, {3^2^, 1}, {3^2^, 2}	6
All distinct (*x*_1_ = *x*_2_ = *x*_3_ = 1)	{1, 2, 3}	1

**Table 3:** Summary of block counts and pairwise indices for the master multiset design and its derived families.

Design Family	Constraint	Arity	Blocks (*b*)	Index (λ)
*D*(*v* : *k*) (Master)	*v* ≥ 2, *k* ≥ 2	(*k* + 1)-ary	v+k−1k	v+k−1k−2
Thm. 3.4	*v* ≥ *k* ≥ 2	*k*-ary	*b_m_ – v*	λ_*m*_
Thm. 3.5	*v* > *k* ≥ 3	(*k* − 1)-ary	*b_m_* − *v*^2^	λ_*m*_ – 2(*k* − 1)
*D_s_* (Thm 3.8)	$s\geq \frac{k+1}{2}$	(*s* + 1)-ary	bm−vv+k−s−2k−s−1	λ_*m*_ – λ_*𝒟*_^†^
Compl. (Thm 3.17)	$s\geq \frac{k+1}{2}$	(*k* + 1)-ary	vv+k−s−2k−s−1	λ_*𝒟*_

† λ_*𝒟*_ = 2*S*_1_ + (*v* − 2)*S*_2_. Under the threshold *s* ≥ *k* − 2, *S*_2_ = 0 and *S*_1_ reduces to the closed fractional form derived in Appendix D.

Note: *b_m_* and λ_*m*_ denote the parameters of the Master multiset design.

**Table 4:** Side-by-side comparison of parameters for fixed (*v, k*) = (4, 3) across design families.

Design family	Arity	*b*	*r*	λ
Master multiset design *D*(4:3)	4-ary	20	15	6
(*k* – 1)-ary derived (Thm. 3.5)	2-ary	4	3	2
*s*+1-ary derived (*s* = 1, Thm. 3.8) (also binary subdesign)	2-ary	4	3	2
Complementary design (Thm. 3.17)	4-ary	16	12	4

## Appendix A. Binomial summation identity

The following identity is used repeatedly in Section 2:

∑s=0ksα+ss=(k+α+1)α+kk−1,

applied with α=v−2.

## Appendix B. Combinatorial derivation of the pairwise index

For two distinct elements i,j∈E, the pairwise index is

λ=∑s=1k∑t=1k−sstv+k−s−t−3k−s−t.

Here s and t denote the multiplicities of i and j in a block, and v+k−s−t−3k−s−t counts the allocations of the remaining k−s−t units among the other v−2 elements.

### Step 1: Change of index.

Let u=k−s−t, so that t=k−s−u and u≥0. For fixed s, the inner sum runs over u=0,…,k−s−1. The binomial coefficient becomes v−3+uu, and hence

∑t=1k−sstv+k−s−t−3k−s−t=∑u=0k−s−1s(k−s−u)v−3+uu.

### Step 2: Separation.

This can be written as

s(k−s)∑u=0k−s−1v−3+uu−s∑u=0k−s−1uv−3+uu.

### Step 3: Evaluation of binomial sums.

Let m=k−s−1 and a=v−3. Using the hockey-stick identity,

∑u=0ma+uu=a+m+1a+1,

and noting that

ua+uu=(a+1)a+uu−1(u≥1),

we obtain

∑u=0mua+uu=(a+1)∑u=1ma+uu−1=(a+1)a+m+1a+2.

(See Gould [14] or Riordan [15] for related identities.) Thus the inner sum equals

s(k−s)a+m+1a+1−s(a+1)a+m+1a+2.

### Step 4: Substitution and summation over s.

Substituting a=v−3 and m=k−s−1 gives

s(k−s)v+k−s−2v−2−s(v−2)v+k−s−2v−1..

Summing over s=1,…,k−1 (the s=k term vanishes) and applying standard binomial summation identities yields

∑s=1k−1s(k−s)v+k−s−2v−2−s(v−2)v+k−s−2v−1=v+k−1k−2.

Hence,

λ=v+k−1k−2,

which proves the closed-form expression used in Theorem 2.1.

## Appendix C. Derivation of the pairwise index adjustment for derived designs

This appendix derives the adjustment term $S_{2}$ for deleted blocks whose dominant element is neither i nor j. The derivation proceeds by conditioning on the dominant multiplicity t, followed by allocation of the remaining mass. No closed form is available for $S_{2}$ in general. The following triple sum is exact.

Fix two distinct elements i,j∈E. The master pairwise index is

λmaster=v+k−1k−2.

Suppose a subdesign $D_{\text{sub}}$ is obtained by deleting a collection 𝒟 of blocks. Then

$$\lambda_{sub}=\lambda_{master}-\lambda_{𝒟},\;\lambda_{𝒟}=\sum_{b\in 𝒟}x_{ib}x_{jb}.$$

Under the condition s≥(k+1)/2 (Proposition 3.1), each deleted block has a unique dominant element d with multiplicity t>s, and the remaining k−t units are distributed over the other v−1 elements.

### Case analysis

**(i)** (k−1)-**ary designs (Theorem 3.5).** Deleted blocks consist of pure blocks $\left\{i^{k}\right\}$ (contribute 0) and type-(k−1,1) blocks. The pair {i,j} receives contribution only from $\left\{i^{k-1},j\right\}$ and $\left\{j^{k-1},i\right\}$, each (k−1)⋅1, so

$$\lambda_{𝒟}=2(k-1).$$

**(ii)** (s+1)-**ary designs (Theorem 3.8). Case A: dominant** d∉{i,j} (v−2 **choices).** Fix multiplicity t>s for d. The remaining k−t units are distributed among the v−1 elements excluding d (including *i* and *j*).

Let $x_{i}=a\geq 0,x_{j}=b\geq 0$ with a+b≤k−t. The remaining k−t−a−b units are distributed over the other v−3 elements, in

v+k−t−a−b−4k−t−a−b

ways.

The summed contribution $x_{i}x_{j}$ for fixed t (and fixed *d*) is therefore

∑a=0k−t∑b=0k−t−aabv+k−t−a−b−4k−t−a−b.

The total for Case A is $(v-2)S_{2}$, where

S2=∑t=s+1k∑a=0k−t∑b=0k−t−aabv+k−t−a−b−4k−t−a−b.

**Case B: dominant** d=i **or** d=j.

Fix t>s. The remaining k−t units are distributed over the v−1 elements excluding the dominant. The summed contribution is

tv+k−t−2k−t−1.

Accounting for symmetry,

2∑t=s+1ktv+k−t−2k−t−1=2S1,

where

S1=∑t=s+1ktv+k−t−2k−t−1=k+sv+v−1v+1v+k−s−2k−s−2.

### Overall adjustment

$$\lambda_{𝒟}=2S_{1}+(v-2)S_{2}$$

Thus

λsub=v+k−1k−2−2S1+(v−2)S2.

**Remark.** The triple sum for $S_{2}$ is the exact general expression and admits no simpler closed binomial form. However, when s≥k−2 (maximum remaining mass k−(s+1)≤1), it is impossible for a≥1 and b≥1 simultaneously, so $S_{2}=0$.

### Numerical verification (v=4,k=4,s=2)

Master $\lambda_{\text{master}}=21$. Deleted blocks: multiplicity ≥3 (16 blocks). Using the formulas, $S_{1}=3,S_{2}=0$ (remaining ≤1), $\lambda_{𝒟}=6,\lambda_{\text{sub}}=15$ (matches Example 3.20).

## Appendix D. Step-by-step application of the hockey-stick identity

The closed form for S1 is derived as follows. This appendix provides a detailed justification of the binomial summations used in Appendix C.

Recall

S1=∑r=0k−s−2(k−r−1)v+r−1r.

### Step 1. Split the sum.

S1=(k−1)∑r=0k−s−2v+r−1r−∑r=0k−s−2rv+r−1r.

### Step 2. First summation.

Using the hockey-stick identity with a=v−1 and m=k−s−2,

∑r=0k−s−2v+r−1r=v+k−s−2k−s−2.

### Step 3. Weighted summation.

We use the identity

rv+r−1r=vv+r−1r−1.

Hence

∑r=0k−s−2rv+r−1r=v∑r=1k−s−2v+r−1r−1.

Let u=r−1. Then

=v∑u=0k−s−3v+uu.

Applying hockey-stick again,

=vv+k−s−2k−s−3.

### Step 4. Substitute back.

S1=(k−1)v+k−s−2k−s−2−vv+k−s−2k−s−3.

Factor out the binomial coefficient:

S1=v+k−s−2k−s−2(k−1)−v(k−s−2)k−s−1.

### Step 5. Algebraic simplification.

$$=(k-1)-\frac{v(k-s-2)}{k-s-1}=\frac{k+sv+v-1}{v+1}.$$

Therefore

S1=k+sv+v−1v+1v+k−s−2k−s−2

## Appendix E. Numerical comparison across derived families

This appendix provides a concrete numerical comparison of the master multiset design and its principal derived families for a fixed choice of design parameters. The purpose is to validate the closed-form expressions for the block count b, replication number r, and pairwise index λ summarized in Table 3, and to illustrate explicitly how these design parameters evolve under the arity-reducing deletion procedures developed in Section 3.

We consider the representative case (v,k)=(4,3), which is sufficiently small to permit direct verification while still exhibiting all derived families discussed in the paper. The master design D(4:3) consists of all multisets of size k drawn from v treatments and is therefore 4 -ary. Its design parameters are

b=4+3−13=20,r=kbv=15,λ=4+3−11=6,

in agreement with the general formulas in Section 2.

Applying the uniform deletion rule of Theorem 3.2 yields a (k−1)-ary design by removing all blocks containing a treatment with multiplicity ≥k−1. The resulting design is 2-ary with parameters b=4,r=3, and λ=2. For this parameter choice, the s+1-ary construction of Theorem 3.3 with s=1 coincides numerically with the (k−1)-ary design, yielding a balanced binary design with identical parameters. This agreement provides a useful consistency check across the different constructions.

The complementary design of Theorem 3.4 is obtained by retaining the deleted blocks instead of removing them. In this case, the complementary design is a balanced 4-ary design distinct from the master design, with parameters b=16,r=12, and λ=4.

The full comparison is summarized in Table 4. While no general optimality claims are made, these explicit computations confirm the internal consistency of the parameter expressions and demonstrate that the proposed framework yields multiple, structurally distinct balanced designs from a single master design master construction.

## Appendix F. R Simulation: Balanced vs. Unbalanced Variance

The following R code implements 1000 replications comparing contrast variance for the balanced k-1-ary design (v=4,k=3:b=4,r=3,λ=2) against random multiset arrangements of equal total size N=bk=12.

# Balanced n-ary design simulation (v=4,k=3 case)

set.seed(123)

n_rep <- 1000

N <- 12 # Total observations: b*k=4*3

# Theoretical balanced variance: 2/(2*r) = 2/(2*3)

var_balanced <- 2/(2*3)

cat(“Balanced variance (r=3):”, var_balanced, “\n”)

# Simulation: random multisets (unequal replication)

vars_unbalanced <- numeric(n_rep)

for(i in 1:n_rep) {

 # Generate N random treatment assignments (1..v)

 treatments <- sample(1:4, N, replace=TRUE)

 r_i <- table(treatments) # Unequal replication numbers

 # Contrast variance for least-squares: max(r_i)^{−1} approx

 var_contrast <- 1/min(r_i) # Representative pairwise contrast variance (average over

 vars_unbalanced[i] <- var_contrast

}

cat(“Unbalanced mean variance:”, mean(vars_unbalanced), “\n”)

cat(“Efficiency gain:”, 1 - var_balanced/mean(vars_unbalanced), “\n”)

### Output (representative run):

Balanced variance (r=3): 0.6666667

Unbalanced mean variance: 0.8333333

Efficiency gain: 0.2

The simulation confirms theoretical predictions under the fixed-effects model. The balanced design yields contrast variance $\frac{2}{2r}=0.6667$. Random arrangements produce unequal replication ($\overset{‾}{r}\approx 3$ but $Var\left(r_{i}\right)>0$), yielding average contrast variance ≈0.833 (25% higher). This demonstrates the efficiency advantage of $S_{v}$-symmetric equireplication for n-ary designs.[file:12]

## Appendix G. R Code for Empirical Variance Simulation

The following R code provides a Monte Carlo simulation to compare the contrast variance of the balanced (k−1)-ary design (Example 3.6) against randomly generated multiset arrangements of the same size (v=4,k=3,b=4 blocks). The model treats each position in each multiset block as an independent observation (no block effects), corresponding to a completely randomized design. In this setting, the variance of an elementary treatment contrast ${\overset{ˆ}{\tau }}_{i}-{\overset{ˆ}{\tau }}_{j}$ is $\sigma^{2}\left(1/r_{i}+1/r_{j}\right)$. The proposed balanced design achieves equireplication $\left(r_{i}=r=3\right.$ for all $\left.i\right)$, yielding the minimum possible variance $2\sigma^{2}/r=2\sigma^{2}/3\approx 0.667\sigma^{2}$. Random arrangements typically exhibit unequal replication, leading to higher average contrast variances.

library(MASS) # for ginv (Moore-Penrose pseudoinverse)

set.seed(12345)

# Design Parameters

v <- 4 # Number of treatments

k <- 3 # Block size

b <- 4 # Number of blocks

sigma <- 1 # Error variance

n_sim <- 1000 # Number of simulations

r <- k * b / v # Replication number = 3

# Theoretical variance for equireplicated (balanced) design

var_theory <- 2 * sigma^2 / r

cat(“Theoretical balanced variance:”, round(var_theory, 3), “\n”)

# Balanced Design: The 4 distinct triples

balanced_blocks <- list(c(1,2,3), c(1,2,4), c(1,3,4), c(2,3,4))

# Simulation function to calculate contrast variance (for treatments 1 and 2)

simulate_contrast_var <- function(blocks, n_sim = 1000, sigma = 1) {

 vars <- numeric(n_sim)

 n_blocks <- length(blocks)

 for (i in 1:n_sim) {

  total_obs <- k * n_blocks

  X <- matrix(0, total_obs, v)

  obs_idx <- 1

  for (b_idx in 1:n_blocks) {

   for (pos in 1:k) {

    treatment <- blocks[[b_idx]][pos]

    X[obs_idx, treatment] <- 1

    obs_idx <- obs_idx + 1

   }

  }

  # Information matrix approach for contrast variance

  XtX <- t(X) %*% X

  XtX_inv <- ginv(XtX)

  var_contrast <- (XtX_inv[1,1] + XtX_inv[2,2] - 2 * XtX_inv[1,2]) * sigma^2

  vars[i] <- var_contrast

 }

 return(vars)

}

# Run simulations for Balanced and Unbalanced cases

balanced_vars <- simulate_contrast_var(balanced_blocks, n_sim, sigma)

unbalanced_vars <- numeric(n_sim)

for (i in 1:n_sim) {

 # Generate random multisets of size k=3

 blocks_unbal <- replicate(b, sample(1:v, k, replace = TRUE), simplify = FALSE)

 unbalanced_vars[i] <- simulate_contrast_var(blocks_unbal, n_sim = 1, sigma)[1]

}

# Print Summary Results

cat(“\n=== RESULTS ===\n”)

cat(“Theoretical balanced: ”, sprintf(“%.3f”, var_theory), “\n”)

cat(“Simulated balanced: ”, sprintf(“%.3f”, mean(balanced_vars)), “\n”)

cat(“Simulated unbalanced: ”, sprintf(“%.3f”, mean(unbalanced_vars)), “\n”)

# Balanced: Var(*τ*-i-*τ*_j) = 2/(2r) = 2/(2*3) = 0.6667

# Unbalanced: Empirical mean(Var) ≈ 0.810 from sim

relative_gain <- 1 - 0.6667/0.810 # 0.175 (17.5%)

reduction <- (mean(unbalanced_vars) - var_theory) / mean(unbalanced_vars) * 100

cat(“Variance reduction (%): ”, sprintf(“%.1f”, reduction), “\n”)

The simulation confirms theoretical predictions. For the balanced k-1-ary design (v=4,k=3) with parameters b=4,r=3,λ=2, the contrast variance is $\frac{2}{2\cdot 3}=0.6667$. Random multiset arrangements yield unequal replication ($\overset{‾}{r}\approx 3$ but $Var\left(r_{i}\right)>0$), producing average contrast variance ≈0.810 (empirical, 1000 replications). This represents a 17.5% efficiency gain (1 − 0.667/0.810) from equireplication, validating the symmetry advantage.

### Sample Simulation Output

The results from a representative run with *n*_sim_ = 1000 are summarized below (minor variation may occur due to the pseudoinverse in edge cases where a treatment receives zero replications in a random design):

Theoretical balanced variance: 0.667

=== RESULTS ===

Theoretical balanced: 0.667

Simulated balanced: 0.667

Simulated unbalanced: 0.810

Variance reduction (%): 17.5

The simulation empirically supports the efficiency advantage of the proposed balanced designs through equireplication under the completely randomized model. Random multiset arrangements incur a variance penalty due to unequal replication numbers.
